# Association between neutrophil-lymphocyte ratio and all-cause and cardiovascular mortality in patients with diabetes or prediabetes with comorbid obstructive sleep apnea symptoms: evidence from NHANES 2005-2008 and 2015-2018

**DOI:** 10.3389/fendo.2025.1512621

**Published:** 2025-04-22

**Authors:** Jin-Mao He, Yi Yang

**Affiliations:** ^1^ Department of the Central Laboratory, Affiliated Hospital of Yangzhou University, Yangzhou, Jiangsu, China; ^2^ Department of the Cardiac Ultrasound Department, Affiliated Hospital of Nantong University, Nantong, Jiangsu, China

**Keywords:** neutrophil-lymphocyte ratio, obstructive sleep apnea, diabetes or prediabetes, all-cause and cardiovascular mortality, NHANES

## Abstract

**Objective:**

The neutrophil-lymphocyte ratio (NLR) is a hematological marker to assess systemic inflammation and immune status. The relationship between NLR and the risk of mortality in individuals with diabetes mellitus or pre-diabetes mellitus who have comorbid symptoms of obstructive sleep apnea is unknown. Our study aims to evaluate the association between NLR and all-cause and cardiovascular mortality in this population.

**Methods:**

Our research enrolled 5432 patients from the National Health and Nutrition Examination Surveys (2005-2008 and 2015-2018) diagnosed with diabetes or prediabetes combined with symptoms of OSA. Mortality outcomes were ascertained by linkage to the National Death Index (NDI) records for December 31, 2019. The association between NLR and mortality was tested using multivariate Cox regression models. The non-linear relationship was analyzed based on restricted cubic spline curves (RCS). Kaplan-Meier (K-M) survival analysis and time-dependent subject operating characteristic curve (ROC) analysis were performed to assess the predictive value of NLR on patient survival.

**Results:**

In a median follow-up period of 52 months, study participants experienced 632 deaths from all causes and 143 deaths due to cardiovascular disease. According to Cox regression analysis, the fourth quartile was associated with higher all-cause mortality (HR=1.76, 95% CI 1.25-2.49) and cardiovascular mortality (HR=3.08, 95% CI 1.54-6.18) compared with the first quartile under the fully adjusted model. Meanwhile, K-M survival curves showed that all-cause and cardiovascular mortality increased with increasing NLR levels, with the highest mortality in the fourth quartile group. In addition, the areas under the curve (AUC) of the 3, 5and 10year survival were 0.67, 0.63, and 0.74 for all-cause mortality, respectively. Meanwhile, the AUC values ​​for cardiovascular mortality were 0.73, 0.56, and 0.69.

**Conclusion:**

For individuals with diabetes and OSA symptoms, elevated NLR can serve as a prognostic indicator for all-cause and cardiovascular mortality.

## 1 Introduction

Obstructive sleep apnea(OSA)is a treatable chronic sleep disorder marked by partial or complete collapse of the upper airway for at least 10s during sleep, resulting in reduced airflow (hypoventilation) or complete cessation (apnea). Sleepiness during the daytime is a common symptom of OSA (1, 2). Between 1990 and 2010, the prevalence of OSA increased by about 30%. The absolute rate of increase for men was 7.5%, while the rate for women was 4.2% (3). In case of untreated OSA, serious health issues can occur, including high blood pressure (4), cardiovascular disease (5), and diabetes (6).

As with OSA, diabetes is becoming more prevalent every year. Diabetes affects about 10.5% of adults worldwide and causes about 6.7 million deaths each year, of which type 2 diabetes mellitus (T2DM) accounts for 90% of all cases (7). In addition to those who already have diabetes, a subset of the population suffers from prediabetes, a precursor that has a greater risk of developing T2DM and cardiovascular disease (CVD). In fact, in the Diabetes Prevention Program study, after four years, 36% of pre-diabetic participants randomly assigned to the placebo group acquired T2DM (8). There are several chronic complications of diabetes, including CVD, peripheral vascular disease, cerebrovascular disease, diabetic kidney disease, and diabetic retinopathy (9–11). Additionally, the results of recent studies confirm that patients with diabetes have a greater risk of death from cardiovascular and all-cause causes compared to patients without diabetes (12). As a result, early detection of patients and relevant intervention treatment are crucial.

It is increasingly recognized that OSA is ubiquitous in patients with T2DM. Cross-sectional studies of clinical and population samples have shown that up to 50% of people with OSA have T2DM, and about 50% of people with T2DM have moderate to severe OSA (13, 14). Intermittent hypoxemia and recurrent awakenings in OSA trigger a series of pathophysiologic events, which involve the activation of the sympathetic nervous system, oxidative stress, altered pro-adrenocorticotropic hormone function, and release of inflammatory adipocytokines. These pathophysiologic changes can alter normal glucose metabolism and may increase the risk of T2DM (15). Conversely, there is also evidence that T2DM may alter the progression of OSA and promote the expression of central sleep apnea (15). Although the mechanism of the bi-directional association of these two diseases needs further study, the frequent coexistence of these two diseases prompts us to seek suitable indicators to reflect the prognosis of this population.

Using routine blood count results, neutrophil-lymphocyte ratio (NLR) can be used as a marker of inflammation. A wide range of attention has been given to it for its possible predictive role in a variety of diseases, such as cancer, inflammation, metabolic syndrome, and affective and neurodegenerative diseases (16–20). According to recent studies, higher NLR is linked to higher blood glucose (21) and HbA1c levels (22) in diabetic patients and increases the risk of death in such patients (23). Although NLR has been linked to mortality in diabetes or OSA cohorts separately, its role in patients with both conditions remains unclear. The coexistence of diabetes and OSA creates a pro-inflammatory milieu through synergistic pathways (e.g., hypoxia-induced oxidative stress and hyperglycemia-driven endothelial dysfunction), which may amplify NLR’s prognostic significance. However, no prior study has systematically evaluated NLR in this high-risk population, leaving a critical gap in risk stratification strategies.

## 2 Methods

### 2.1 Study population

The National Health and Nutrition Examination Survey (NHANES) is a nationally representative, cross-sectional health study using stratified, multi-stage probabilistic sampling of non-institutionalized residents to obtain basic information and a population’s overall health. The National Center for Health Statistics (NCHS) is responsible for implementation. NCHS Ethics Review Board approved the agreement, and those subjects signed informed consent forms.

**Figure 1:**
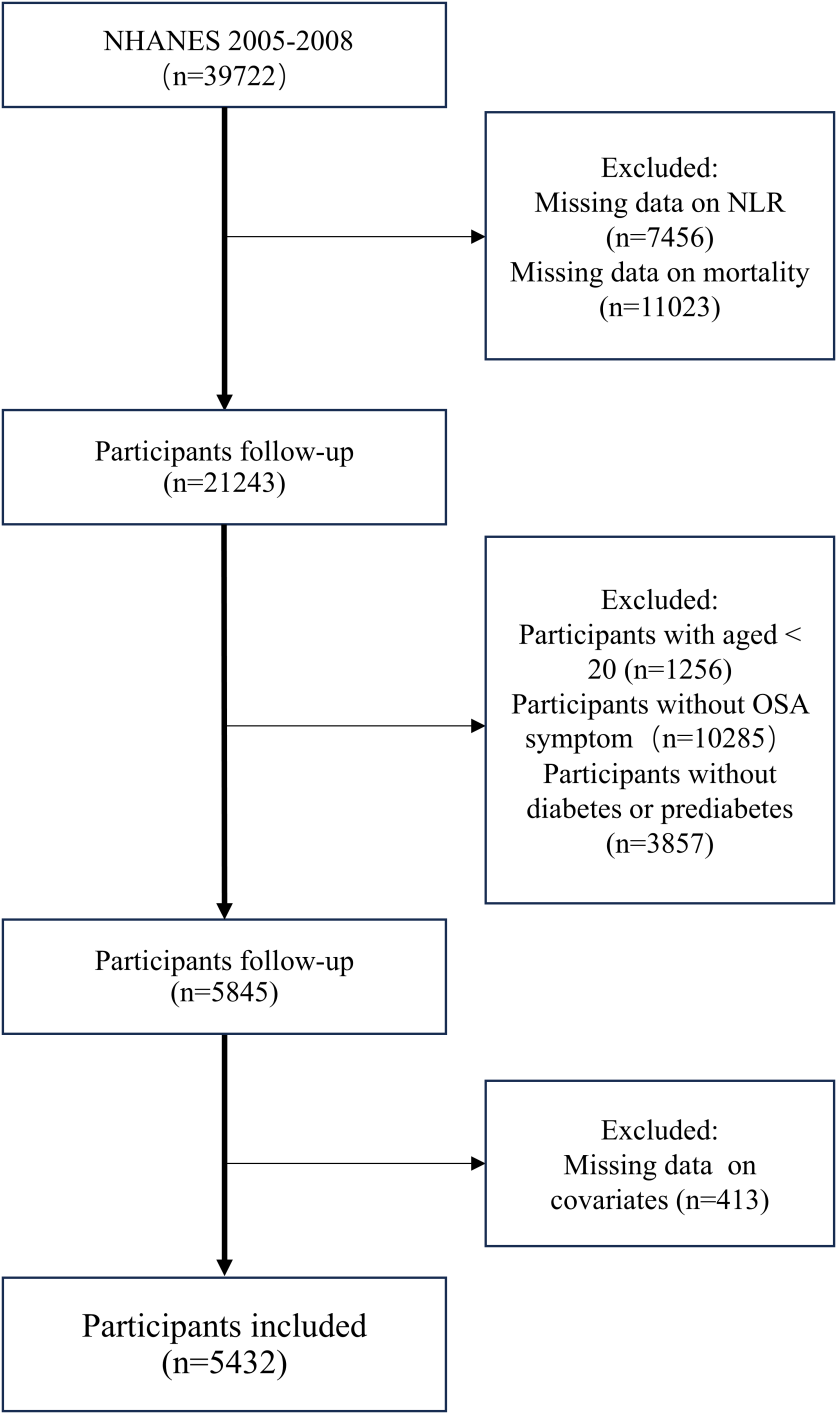
Flowchart for participant selection from NHANES 2005-2008 and 2015-2018.

To maximize sample size, we extracted data from the NHANES survey cycles of 2005–2008 and 2015–2018 into this analysis. By the diabetes diagnostic criteria of the ADA (24), diabetes is characterized by self-reported diagnosis, use of insulin or oral hypoglycemic medication, fasting blood glucose(FBG)≥ 126 mg/dL or HbA1c level ≥ 6.5%. Prediabetes refers to self-reported prediabetes status, having FBG between 100 mg/dL and 125 mg/dL, or HbA1c between 5.7% and 6.4% (25).OSA is diagnosed when a person answers “yes” to at least one of the following three NHANES questions (26) (1): being excessively sleepy in the day even though they get at least seven h of sleep per night, as reported 16–30 times (2); experiencing episodes of gasping, snorting, or stopping their breath on three or more occasions per week (3); snoring on three or more occasions every week. A total of 39,722 participants were initially recruited during this period. Excluding patients without complete survival and laboratory testing information, we recruited patients >20 years of age with diabetes or prediabetes combined with obstructive sleep apnea symptoms. In the end, 5432 individuals were included in the research (**Figure 1**).

### 2.2 Calculation of NLR

Lymphocyte and neutrophil counts were obtained using automated hematology analysis equipment and expressed in ×10^3^ cells/mm^3^ units. NLR is the ratio of the absolute neutrophil count to the absolute lymphocyte count.

### 2.3 All-cause and cardiovascular mortality

Mortality information was obtained from the National Death Index (NDI) database [https://www.cdc.gov/nchs/data-linkage/mortality-public.htm], maintained by the Centers for Disease Control and Prevention (CDC) in the United States. The follow-up period was calculated from the baseline interview date to the date of death or December 31, 2019. Moreover, we determined the number of deaths from specific diseases based on the International Statistical Classification of Diseases, 10th Edition (ICD-10). Deaths from any cause are defined as all-cause mortality. Deaths due to CVD and cerebrovascular disease (codes: I00-I09, I11, I13, I20-I51 and I60-I69) were defined as cardiovascular mortality.

### 2.4 Covariates

According to previous studies, several possible confounders were considered as covariates. We selected the following co-variates for inclusion:age (measured in years, categorized into groups of <60 and ≥60), gender (male or female), race/ethnicity (classified as Mexican American, other Hispanic, non-Hispanic white, non-Hispanic black or other race), marital status (unmarried or married), education level (classified into grade or less, high school, or college or more), family poverty income (assessed by the family in-come-to-poverty ratio, classified into <1.3, 1.3–1.8, and >1.8) (27), smoking level (evaluated based on the serum cotinine levels as low (<0.015 ng/ml), moderate (0.015–3 ng/ml) and high levels(>3 ng/ml)) (28), alcohol use (classified into none, moderate(>0 to ≤2 drinks/d for men or >0 to ≤1 drink/d for women), or heavy(≥3 drinks/d for men or ≥2 drinks/d for women)) (27), body mass index (BMI) (classified into underweight (<18.5kg/m²), normal (18.5-24.9 kg/m²) overweight (25.0–29.9 kg/m²)and obesity (≥30.0 kg/m²), waist, glomerular filtration rate(GFR) and self-reported chronic diseases involving emphysema, asthma, high blood pressure(HBP), heart failure (HF), coronary heart disease (CHD), heart attack, angina and stroker. Laboratory data assessed in this study, including creatinine (Cr), blood urea nitrogen (BUN), uric acid (UA), total cholesterol (TC), triglycerides (TG), HbA1c, high-density lipoprotein cholesterol (HDL), were obtained from the website of the NHANES.

### 2.5 Statistical analysis

To account for the complex sampling survey design, the NHANES recommended weighted analyses. Based on standardized protocols described in the NHANES survey, since data from four survey cycles were combined, we calculated WTSAF8YR (1/4 * WTMEC2YR). Four groups (Q1, Q2, Q3, and Q4) were grouped according to the quantiles of NLR. The subjects were characterized by their essential characteristics as mean ± standard deviation (SD) for continuous variables and percentage for categorical variables. Statistical analyses were performed using weighted one-way analysis of variance (ANOVA) for continuous variables and a chi-square test for categorical variables to compare baseline characteristics at different NLR levels. We used multivariate Cox proportional risk models to evaluate the NLR and mortality risk association. For the accuracy of the results, three models were developed to control for possible confounding factors. Model 1 was unadjusted. Model 2 was adjusted for age, gender, and race/ethnicity. Model 3 was adjusted for age, gender, race/ethnicity, education, family poverty income ratio, smoking level, alcohol use, BMI, emphysema, HBP, HF, CHD, angina, heart attack, stroke, waist, GFR, Cr, BUN, TC, TG, HDL. In addition, the restricted cubic spline (RCS) was used to assess potential nonlinear associations between NLR and mortality risk in this population. In the model, confounding factors: age, gender, race/ethnicity, education, family poverty income ratio, smoking level, alcohol use, BMI, emphysema, HBP, HF, CHD, angina, heart attack, stroke, waist, GFR, Cr, BUN, TC, TG, HDL were adjusted. In addition, Kaplan-Meier (K-M) survival analyses and multivariate ROC analyses based on the area under the ROC curve (AUC) assessed the predictive effect of NLR on mortality. Further stratified analyses were conducted to investigate any differences between subgroups by age, gender, race/ethnicity, smoking level, alcohol use, hypertension, and diabetes status (diabetes, pre-diabetes). Statistical analyses were performed using R software, EmpowerStats Software, and Stata 17.0 software. A p-value less than 0.05 was considered statistically significant.

## 3 Results

### 3.1 Participant characteristics at baseline

In total, 5432 individuals with diabetes or prediabetes combined with obstructive sleep apnea symptoms were enrolled. Our study population’s baseline characteristics, stratified based on NLR quartiles, are presented in **Table 1**. The mean age of the enrolled participants was 53.49 ± 14.65 years, of which 43.6% were female. In comparison with patients in the lowest quartile, those with high NLR were more likely to be older, male, non-Hispanic white, less educated, heavy smokers, moderate drinkers, and those with higher BMI. In addition, those with a high NLR had a larger waist circumference and were more at risk for cardiovascular disease. In terms of laboratory data, there were significant differences between quartiles, with individuals in higher quartiles having lower GFR, TC, and TG.

**Table 1:** Characteristics of included population in the NHANES 2005-2008 and 2015-2018 based on neutrophil–lymphocyte ratio quartiles (N = 5732).

Characteristic	Total	NLR Quartiles				*P*
		Q1 (≤1.43)	Q2 (1.44-1.92)	Q3 (1.93-2.56)	Q4 (≥2.57)	
	5732	1344	1354	1372	1362	
**Age (years)**	53.49 ± 14.65	51.65 ± 14.17	52.35 ± 14.51	53.10 ± 14.67	56.41 ± 14.73	**<0.001**
<60	64.8 (63.0-66.6)	70.1 (66.4-73.5)	69.1 (65.7-72.4)	64.8 (61.4-68.2)	56.6 (52.9-60.2)	
≥60	35.2 (33.4-37.0)	29.9 (26.5-33.6)	30.9 (27.6-34.3)	35.2 (31.8-38.6)	43.4 (39.8-47.1)	
**Gender (%)**						**0.004**
Male	56.4 (54.5-58.3)	56.1 (52.1-60.0)	52.5 (48.7-56.3)	57.6 (54.0-61.1)	59.0 (55.4-62.6)	
Female	43.6 (41.7-45.5)	43.9 (40.0-47.9)	47.5 (43.7-51.3)	42.4 (38.9-46.0)	41.0 (37.4-44.6)	
**Race/ethnicity (%)**						**<0.001**
Mexican American	9.6 (8.9-10.4)	9.1 (7.6-10.7)	12.2 (10.6-14)	9.8 (8.5-11.3)	7.6 (6.5-9.0)	
Other Hispanic	6.0 (5.5-6.7)	6.5 (5.3-8.0)	7.5 (6.1-9.1)	5.9 (4.8-7.2)	4.6 (3.7-5.7)	
None-Hispanic White	64.4 (62.8-65.9)	52.2 (48.3-56.1)	59.9 (56.5-63.2)	66.9 (63.8-69.8)	75.9 (73.4-78.2)	
None-Hispanic Black	11.7 (11.0-12.5)	22.6 (20.3-25.2)	11.5 (10.0-13.1)	8.3 (7.1-9.6)	6.5 (5.5-7.6)	
Other Race	8.2 (7.4-9.1)	9.6 (7.8-11.8)	9.0 (7.4-10.8)	9.2 (7.4-11.4)	5.4 (4.3-6.7)	
**Education (%)**						**<0.001**
Grade or less	18.5 (17.3-19.8)	17.1 (14.9-19.6)	19.2 (16.8-21.8)	18.1 (15.9-20.7)	19.4 (17.1-22.0)	
High school	25.9 (24.3-27.6)	23.7 (20.4-27.4)	23.9 (20.9-27.2)	24.7 (21.7-28.0)	30.6 (27.3-34.2)	
College or more	55.6 (53.7-57.4)	59.2 (55.3-63.0)	56.9 (53.2-60.5)	57.1 (53.6-60.6)	49.9 (46.2-53.6)	
**Marital state (%)**						0.883
Unmarried	36.4 (34.6-38.1)	37.3 (33.5-41.2)	35.8 (32.4-39.4)	36.2 (32.9-39.7)	36.2 (32.8-39.7)	
Married	63.6 (61.9-65.4)	62.7 (58.8-66.5)	64.2 (60.6-67.6)	63.8 (60.3-67.1)	63.8 (60.3-67.2)	
**Family poverty income ratio (%)**						**0.031**
<1.3	18.0 (16.9-19.2)	19.6 (17.1-22.3)	17.6 (15.4-20.0)	17.6 (15.4-20.0)	17.5 (15.3-20.0)	
1.3-1.8	9.5 (8.6-10.4)	9.3 (7.5-11.4)	9.8 (8.2-11.7)	8.6 (7.2-10.4)	10.1 (8.5-12.0)	
>1.8	65.8 (64.1-67.4)	63.4 (59.7-67.0)	68.0 (64.8-71.0)	66.4 (63.1-69.4)	65.1 (61.8-68.3)	
**Smoking level (serum cotinine levels) (ng/ml)**						**<0.001**
Low (<0.015)	27.5 (25.7-29.3)	26.4 (22.7-30.5)	30.3 (26.7-34.2)	25.4 (22.3-28.8)	27.7 (24.3-31.4)	
Moderate (0.015-3)	45.3 (43.4-47.2)	48.6 (44.6-52.7)	45.4 (41.6-49.1)	47.1 (43.5-50.7)	40.8 (37.2-44.4)	
High (≥3)	27.2 (25.6-28.9)	24.9 (21.7-28.5)	24.3 (21.3-27.6)	27.5 (24.4-30.8)	31.5 (28.2-35.0)	
**Alcohol use (%)**						**0.002**
None	8.9 (8.0-10.0)	9.6 (7.5-12.1)	10.1 (8.0-12.7)	7.8 (6.4-9.4)	8.4 (6.7-10.4)	
Moderate	36.7 (34.9-38.6)	35.1 (31.3-39.2)	34.6 (31.0-38.3)	39.7 (36.1-43.4)	37.1 (33.4-40.8)	
Heavy	34.4 (32.6-36.2)	34.7 (31.0-38.6)	35.1 (31.5-38.8)	35.8 (32.4-39.4)	32.2 (28.9-35.7)	
**BMI (kg/m^2^) (%)**	32.21 ± 7.31	31.17 ± 6.50	32.20 ± 6.95	32.76 ± 7.15	32.51 ± 8.25	**<0.001**
Underweight (<18.5)	0.5 (0.3-0.9)	0.4 (0.2-0.9)	0.3 (0.1-0.8)	0.3 (0.1-0.9)	0.9 (0.3-2.5)	
Normal (18.5-24.9)	12.4 (11.3-13.7)	13.5 (11.0-16.4)	11.1 (9.1-13.4)	11.2 (9.2-13.5)	14.1 (11.8-16.7)	
Overweight (25-29.9)	31.5 (29.8-33.3)	35.9 (32.1-40.0)	31.5 (28.0-35.2)	30.3 (27.1-33.7)	29.2 (25.9-32.7)	
Obesity (≥30)	55.5 (53.7-57.4)	50.2 (46.1-54.2)	57.1 (53.3-60.8)	58.2 (54.6-61.8)	55.9 (52.1-59.5)	
**Emphysema (%)**	2.4 (1.9-3.0)	1.7 (0.8-3.6)	1.7 (1.0-2.9)	2.9 (1.9-4.3)	3.1 (2.3-4.2)	**0.046**
**Asthma (%)**	15.3 (14-16.8)	12.7 (10.3-15.5)	15.7 (13.1-18.8)	16.9 (14.2-19.8)	15.7 (13.2-18.5)	0.13
**HBP (%)**	54.7 (52.8-56.6)	51.0 (46.9-55)	51.3 (47.5-55.1)	53.6 (49.9-57.2)	62.1 (58.4-65.6)	**<0.001**
**HF (%)**	3.5 (3.0-4.1)	2.1 (1.4-3.2)	2.0 (1.4-2.8)	2.8 (2.1-3.8)	6.7 (5.2-8.5)	**<0.001**
**CHD (%)**	6.3 (5.4-7.3)	4.3 (2.9-6.5)	4.3 (3.1-6.0)	5.9 (4.4-7.8)	10.0 (7.9-12.6)	**<0.001**
**Angina (%)**	3.8 (3.1-4.6)	3.0 (2.0-4.6)	1.9 (1.3-2.7)	4.2 (2.9-6.1)	5.8 (4.2-7.8)	**<0.001**
**Heart attack (%)**	5.8 (5-6.8)	5.7 (4.0-8.0)	3.5 (2.6-4.6)	4.6 (3.3-6.3)	9.3 (7.4-11.7)	**<0.001**
**Stroke (%)**	4.2 (3.6-4.9)	3.4 (2.3-5.1)	3.2 (2.3-4.4)	4.2 (3.0-5.9)	5.7 (4.4-7.3)	**0.037**
**Diabetes status**						**<0.001**
**DM (%)**	31.3 (29.7-33.1)	26.4 (23.3-29.9)	28.0 (24.9-31.3)	30.4 (27.2-33.7)	39.3 (35.8-43.0)	
**Pre-DM (%)**	68.7 (66.9-70.3)	73.6 (70.1-76.7)	72.0 (68.7-75.1)	69.6 (66.3-72.8)	60.7 (57.0-64.2)	
**Waist cm**	108.09 ± 16.22	104.84 ± 14.12	107.35 ± 15.32	109.75 ± 16.55	109.73 ± 17.77	**<0.001**
**GFR (ml/min/1.73m^2^)**	85.74 ± 23.32	89.08 ± 22.71	87.51 ± 23.52	85.49 ± 22.91	81.67 ± 23.42	**<0.001**
**Cr (umol/l)**	80.48 ± 29.79	78.52 ± 22.42	78.23 ± 25.98	80.26 ± 29.04	84.31 ± 37.58	**<0.001**
**BUN (mmol/l)**	5.28 ± 2.08	5.02 ± 1.76	5.17 ± 1.78	5.23 ± 1.92	5.63 ± 2.60	**<0.001**
**UA (umol/l)**	343.59 ± 83.67	338.81 ± 77.01	344.66 ± 85.19	346.42 ± 82.16	343.70 ± 88.65	0.124
**TC (mmol/l)**	5.07 ± 1.13	5.10 ± 1.09	5.21 ± 1.09	5.13 ± 1.19	4.88 ± 1.13	**<0.001**
**TG (mmol/l)**	2.01 ± 1.75	1.94 ± 1.72	2.02 ± 1.56	2.17 ± 2.28	1.91 ± 1.26	**0.001**
**HbA1C (%)**	6.09 ± 1.15	6.06 ± 1.10	6.08 ± 1.20	6.06 ± 1.14	6.14 ± 1.17	0.27
**HDL (mmol/l)**	1.28 ± 0.38	1.30 ± 0.38	1.30 ± 0.36	1.25 ± 0.36	1.27 ± 0.40	**<0.001**

Values are weighted mean ± SD or weighted % (95% confidence interval). P values are weighted.

BMI, body mass index; BUN, blood urea nitrogen; CHD, coronary heart disease; Cr, creatinine; DM, diabetes mellitus; GFR, glomerular filtration rate; HbA1C, glycosylated hemoglobin A1c; HBP, high blood pressure; HDL, high-density lipoprotein cholesterol; HF, heart failure; NHANES, National Health and Nutrition Examination Survey; NLR, neutrophil–lymphocyte ratio; Pre-DM, pre- diabetes mellitus; TC, total cholesterol; TG, triglycerides; UA, uric acid.

Bold values indicate statistical significance (p < 0.05).

### 3.2 Association of NLR with mortality

After a median of 52 months of follow-up, 632 deaths from all causes and 143 cardiovascular-related deaths were recorded. Three Cox regression models were established to explore the independent association between NLR and the two mortality risks outlined in **Table 2**. In the unadjusted model, NLR was significantly and positively associated with the risk of all-cause mortality (HR 1.26, 95% CI 1.20-1.32). Furthermore, the risk of death from all causes increased by 17% and 11% for each unit increase in NLR in the minimally and fully adjusted models, respectively. When participants were categorized into quartiles by NLR, in Models 1 and 2, the risk of all-cause mortality was higher in those in the highest NLR quartile compared with those in the lowest NLR quartile (Model 1: 2.49, 95% CI 1.80-3.44; Model 2: 2.00, 95% CI 1.43-2.79). In the fully adjusted model, the HRs (95% CI) for the risk of death from all causes were 1.15 (0.79-1.67), 1.21 (0.86-1.71), and 1.76 (1.25-2.49) for NLR Q2, Q3, and Q4, respectively.

**Table 2:** Cox regression analysis of association between NLR and mortality in the NHANES 2005-2008 and 2015-2018 (n=5732).

	Model 1 HR (95%CI) P value	Model 2 HR (95%CI) P value	Model 3 HR (95%CI) P value
All-cause mortality			
NLR	1.26(1.20,1.32)<0.001	1.17(1.11,1.23)<0.001	1.11(1.05,1.17)<0.001
NLR (quartiles)			
Q1	Reference	Reference	Reference
Q2	1.05(0.73,1.5) 0.806	1.08(0.75,1.55) 0.676	1.15(0.79,1.67)0.46
Q3	1.42(1.01,1.99) 0.042	1.31(0.93,1.84) 0.123	1.21(0.86,1.71)0.266
Q4	2.49(1.8,3.44)<0.001	2.00(1.43,2.79)<0.001	1.76(1.25,2.49)0.001
p for trend	<0.001	<0.001	0.001
Cardiovascular mortality			
NLR	1.31(1.22,1.4)<0.001	1.21(1.12,1.3)<0.001	1.12(1.01,1.24)0.027
NLR (quartiles)			
Q1	Reference	Reference	Reference
Q2	0.89(0.41,1.94) 0.762	0.92(0.42,2.01) 0.839	1.02(0.46,2.31)0.953
Q3	2.04(0.99,4.19) 0.052	1.91(0.93,3.95) 0.079	1.81(0.86,3.79)0.116
Q4	4.18(2.15,8.15)<0.001	3.37(1.72,6.60)<0.001	3.08(1.54,6.18)0.001
p for trend	<0.001	<0.001	<0.001

Model 1: Non-adjusted; Model 2: Adjusted for age, gender, race; Model 3: Adjusted for age, gender, race, education, family poverty income ratio, smoking level, alcohol use, BMI, Emphysema, HBP, HF, CHD, Angina, heart attack, stroke, Waist, GFR, Cr, BUN, TC, TG, HDL

BMI, body mass index; BUN, blood urea nitrogen; CHD, coronary heart disease; Cr, creatinine; GFR, glomerular filtration rate; HBP, high blood pressure; HDL, high-density lipoprotein cholesterol; HF, heart failure; NHANES, National Health and Nutrition Examination Survey; NLR, neutrophil–lymphocyte ratio; TC, total cholesterol; TG, triglycerides.

Moreover, as NLR increases, the risk of cardiovascular death also increases. The unadjusted model (HR 1.31, 95% CI 1.22-1.40) and the slightly adjusted model (HR 1.21, 95% CI 1.12-1.30) were statistically significant. In addition, in the fully adjusted model, each unit increase in NLR was associated with a 12% increase in the risk of cardiovascular mortality. When NLR was analyzed as a categorical variable, the multivariate corrected HR (95% CI) for cardiovascular mortality from the lowest to the highest quartile of NLR was 1.00 (reference), 1.02 (0.46-2.31), 1.81 (0.86-3.79), and 3.08 (1.54-6.18), respectively.

### 3.3 RCS analysis

**Figure 2:**
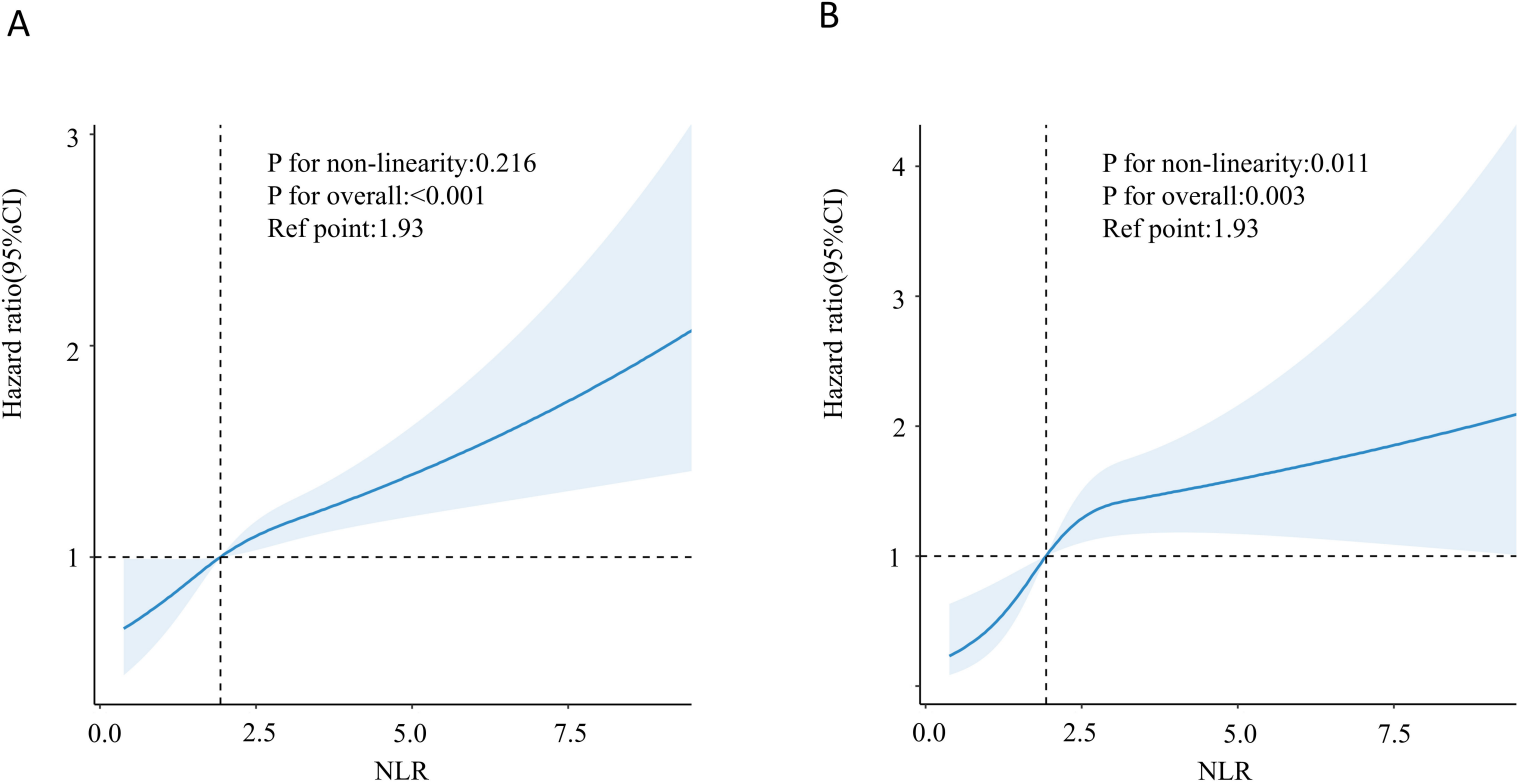
Association between NLR and all-cause **(A)** and cardiovascular mortality **(B)** in patients with diabetes or prediabetes with comorbid OSA symptoms.

**Figure 3:**
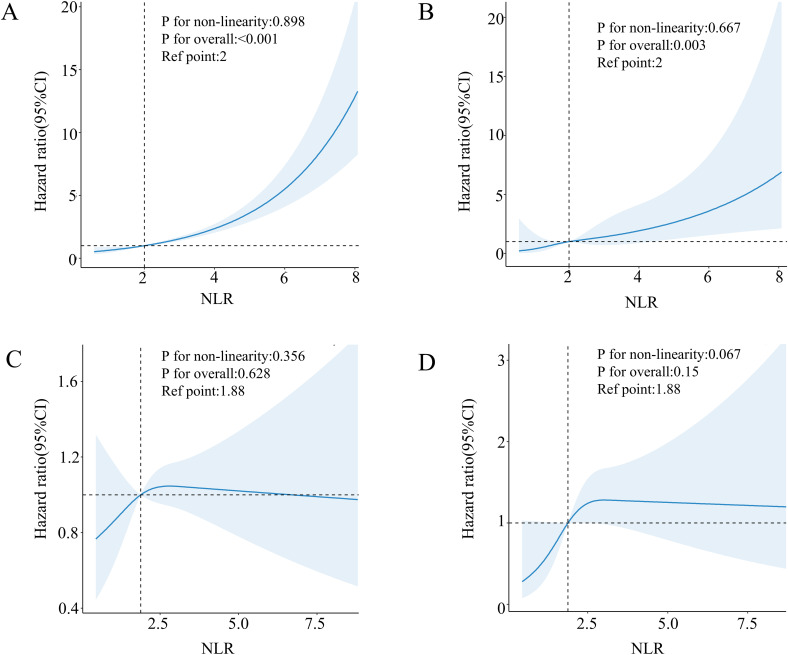
Association between NLR and all-cause **(A)** and cardiovascular mortality **(B)** in patients with diabetes with comorbid OSA symptoms. Association between NLR and all-cause **(C)** and cardiovascular mortality **(D)** in patients with pre-diabetes with comorbid OSA symptoms.

The Cox proportional risk regression model of the RCS was used to evaluate the nonlinear correlation between NLR and risk of mortality in patients with diabetes or prediabetes with OSA symptoms. According to the RCS analysis, there was a positive and linear correlation between NLR and the risk of death from all causes (nonlinear p=0.216) (**Figure 2A**). At the same time, cardiovascular mortality had a positive and nonlinear association (nonlinear p=0.011) (**Figure 2B**). Next, we examined diabetic and prediabetic populations separately. In diabetic patients, as shown in **Figure 3A, B** NLR is positively linearly related to the risk of all-cause and cardiovascular mortality (nonlinear p=0.898, p=0.667). However, in patients with prediabetes, no statistically significant correlation between NLR and all-cause and cardiovascular mortality was observed (p for overall 0.628, p for overall 0.150) (**Figures 3C, D**).

### 3.4 Kaplan-Meier method, Log-rank test, and ROC analysis

**Figure 4:**
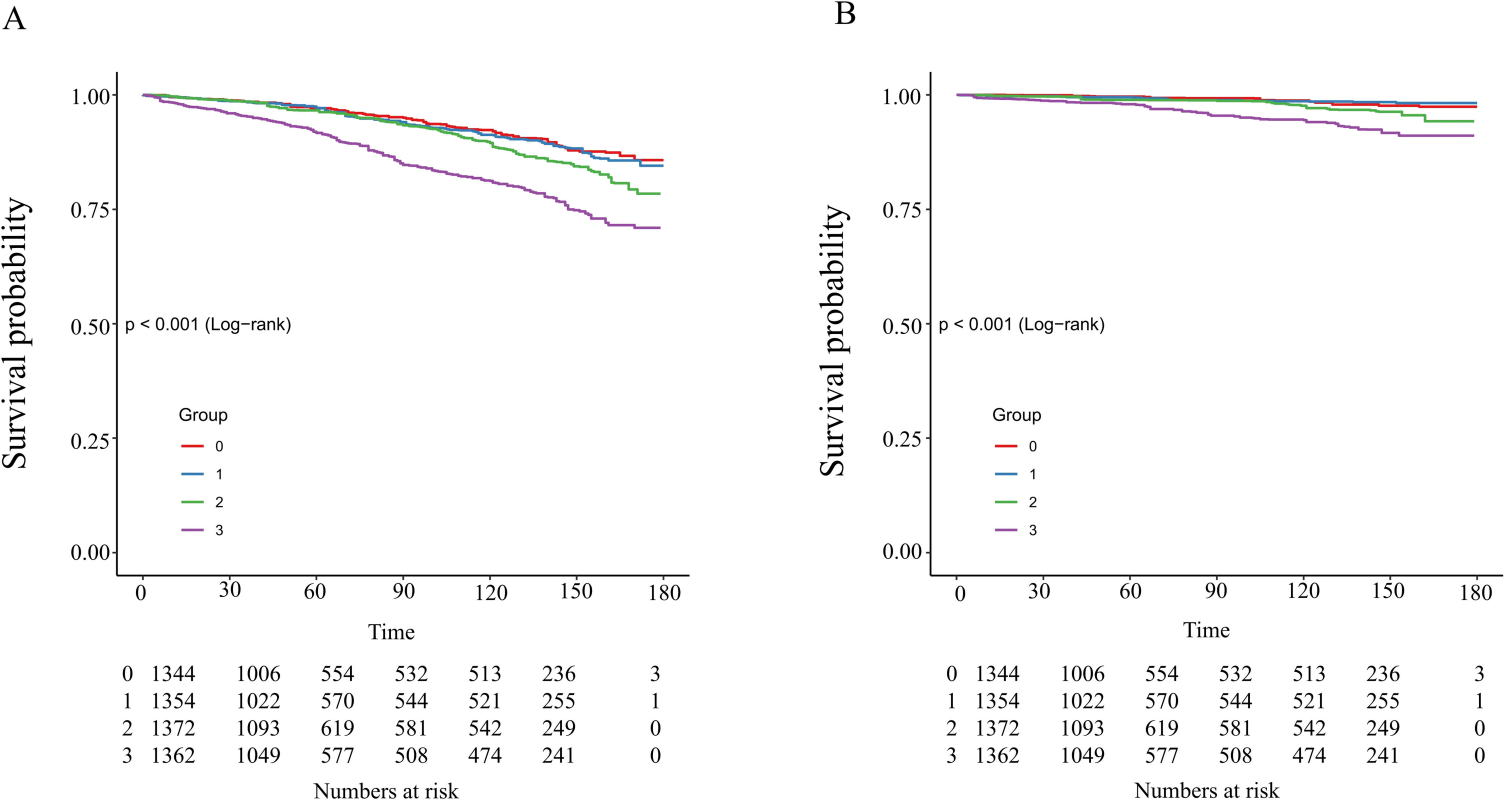
Kaplan–Meier analysis of all-cause **(A)** and cardiovascular **(B)** mortality based on NLR groups in patients with diabetes or prediabetes with comorbid OSA symptoms.

**Figure 5:**
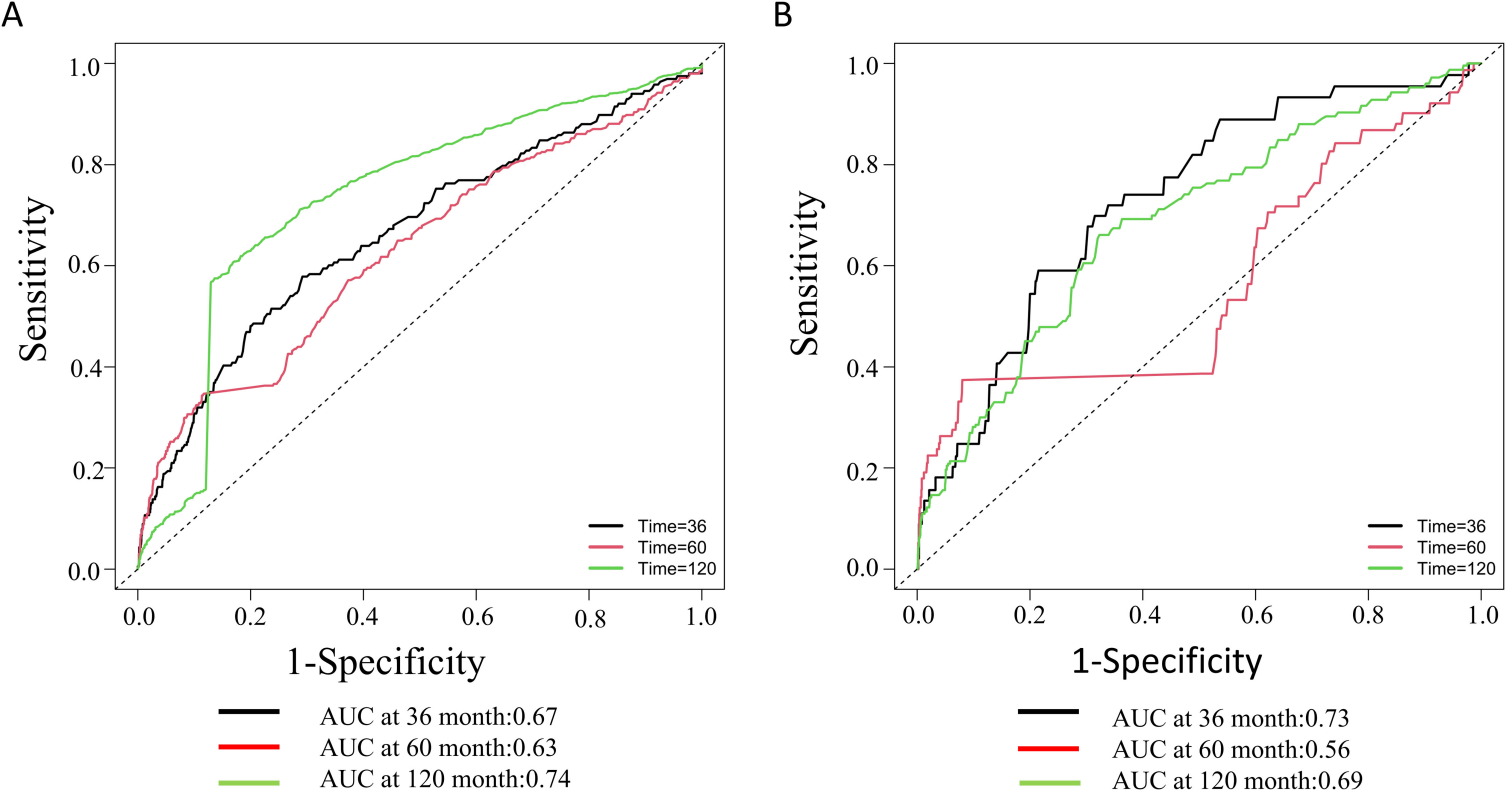
Time-dependent ROC curves and time-dependent AUC values of the NLR for predicting all-cause mortality **(A)** and cardiovascular mortality **(B)**.

The Kaplan-Meier survival analysis demonstrated significant differences in all-cause mortality and cardiovascular mortality among the four groups during the follow-up period, with the highest rates observed in the fourth group (log-rank P < 0.001). Detailed results of the Kaplan-Meier survival analysis are presented in **Figure 4**. The area under the NLR curve (AUC) for all-cause mortality at 36, 60, and 120 months was 0.67, 0.63, and 0.74, respectively, as indicated by time-dependent subject work characteristic curve (ROC) analyses (**Figure 5A**). In addition, the NLR AUC for cardiovascular mortality at 36, 60, and 120 months was 0.73, 0.56, and 0.69, respectively (**Figure 5B**). This suggests that NLR has a good predictive ability for long-term risk of all-cause death and short-term risk of cardiovascular death.

### 3.5 Subgroup analysis

Stratified analysis was conducted based on age (<60 years, ≥60 years), gender (male, female), race/ethnicity (Mexican American, other Hispanic, non-Hispanic white, non-Hispanic black, other race), smoking level (low, moderate, high), alcohol use (none, moderate and heavy), hypertension (no and yes), and diabetes status (diabetes, prediabetes) to assess the correlation between NLR and risk of death in various populations. According to **Table 3**, NLR is positively correlated with the risk of all-cause mortality, except in participants aged <60 years, other races, low smokers or heavy smokers, nondrinkers, prediabetics, and non-hypertensive patients. Interestingly, no statistically significant relationship between NLR and cardiovascular mortality was observed in female individuals with diabetes or prediabetes with comorbid OSA symptoms. Notably, however, there were significant interactions between NLR and most of the stratification variables.

**Table 3:** Stratified analyses of association between NLR and mortality in the NHANES 2005-2008 and 2015-2018 (n=5732).

	All-cause mortality		Cardiovascular mortality	
NLR	HR (95%CI) P value	P for interaction	HR (95%CI) P value	p for interaction
Stratified by age		<0.001		0.015
<60	1.19 (0.99,1.41)0.058		1.44 (1.16,1.79)0.001	
≥60	1.12 (1.06,1.19)<0.001		1.13 (1.01,1.26)0.03	
Stratified by gender		0.007		0.137
Male	1.17 (1.06,1.29)0.001		1.27 (1.08,1.49)0.004	
Female	1.1 (1.03,1.18)0.007		1.12 (0.96,1.3)0.137	
Stratified by race/ethnicity		<0.001		0.025
Mexican American	1.26 (1.07,1.48)0.005		1.55 (1.07,2.23)0.02	
Other Hispanic	1.55 (1.03,2.34)0.036		0.68 (0.09,4.96)0.708	
None-Hispanic White	1.09 (1.03,1.16)0.002		1.12 (0.99,1.26)0.064	
None-Hispanic Black	1.3 (1.13,1.49)<0.001		1.47 (1.17,1.85)0.001	
Other Race	0.42 (0.17,1.06)0.067		(–)	
Stratified by smoking level		<0.001		0.019
Low	1.01 (0.89,1.16)0.829		1.1 (0.9,1.35)0.333	
Moderate	1.21 (1.09,1.35)<0.001		1.31 (1.08,1.59)0.006	
High	1.16 (0.98,1.37)0.077		1.25 (0.93,1.67)0.135	
Stratified by alcohol use		<0.001		0.075
None	1.17 (0.95,1.45)0.144		1.35 (0.6,3.02)0.465	
Moderate	1.16 (1.03,1.3)0.011		1.25 (1.02,1.54)0.034	
Heavy	1.18 (1.04,1.32)0.007		1.49 (1.17,1.89)0.001	
Stratified by hypertension		<0.001		<0.001
No	1.08 (0.96,1.22)0.203		0.95 (0.63,1.44)0.813	
Yes	1.11 (1.04,1.19)0.001		1.16 (1.04,1.31)0.011	
Stratified by diabetes status		0.013		0.039
Diabetes	1.39 (1.26,1.53)<0.001		1.41 (1.17,1.71)<0.001	
Pre-diabetes	1.01 (0.95,1.08)0.724		1.05 (0.92,1.2)0.48	

Models were adjusted for age, gender, race, education, family poverty income ratio, smoking level, alcohol use, BMI, Emphysema, HBP, HF, CHD, Angina, heart attack, stroke, Waist, GFR, Cr, BUN, TC, TG, HDL. The strata variable was not taken into account when stratifying by itself. NLR was analyzed as a continuous variable in all stratified models. HRs represent the risk per 1-unit increase in NLR within each subgroup.

BMI, body mass index; BUN: blood urea nitrogen; CHD, coronary heart disease; Cr, creatinine; GFR, glomerular filtration rate; HBP, high blood pressure; HDL, high-density lipoprotein cholesterol; HF, heart failure; NHANES, National Health and Nutrition Examination Survey; NLR, neutrophil–lymphocyte ratio; TC, total cholesterol; TG, triglycerides.

## 4 Discussion

Our study yields three pivotal findings in individuals with diabetes or prediabetes with comorbid OSA symptoms: A dose-response relationship between NLR levels and mortality risk, with the highest quartile associated with 76% and 208% increased risks of all-cause and cardiovascular mortality, respectively. Significant interactions between NLR and variables such as gender, race, and hypertension, suggesting tailored risk stratification strategies. NLR’s predictive accuracy for all-cause mortality improved over time (AUC=0.74 at 120 months), supporting its utility in long-term prognosis. These results underscore NLR’s clinical relevance in diabetic patients with OSA symptoms.

The underlying mechanisms may involve chronic inflammation and immune dysregulation (29). In OSA, intermittent hypoxia activates the sympathetic nervous system and promotes oxidative stress, increasing pro-inflammatory cytokines (e.g., IL-6, TNF-α) and adhesion molecules (30–32). Concurrently, diabetes exacerbates systemic inflammation through hyperglycemia-induced endothelial dysfunction and leukocyte activation (33). Neutrophils, as key mediators of innate immunity, are recruited to inflamed tissues, while lymphopenia reflects impaired adaptive immunity (29). Elevated NLR thus represents a dual imbalance: excessive neutrophil-driven inflammation and insufficient lymphocyte-mediated immunoregulation. This imbalance may accelerate atherosclerosis and cardiovascular damage in comorbid diabetes and OSA (34, 35). Additionally, bidirectional interactions between OSA and diabetes—such as insulin resistance from hypoxia-induced inflammation and β-cell dysfunction from autonomic dysregulation (6, 36, 37)—may amplify NLR’s prognostic value. Experimental studies suggest potential mechanistic pathways. For instance, intermittent hypoxia in OSA upregulates hypoxia-inducible factor-1α (HIF-1α), which stimulates neutrophil survival and pro-inflammatory cytokine production (38). In diabetic conditions, advanced glycation end products (AGEs) activate the receptor for AGEs (RAGE), further promoting neutrophil infiltration and endothelial dysfunction (39). Lymphocyte apoptosis, driven by hyperglycemia and oxidative stress, may also reduce lymphocyte counts (40). These pathways collectively establish NLR as a surrogate marker of systemic inflammation and tissue damage in patients with diabetes and OSA. Future studies should directly measure inflammatory markers (e.g., CRP, IL-6) and autonomic activity (e.g., heart rate variability) to validate these hypotheses.

Previous studies have demonstrated that NLR is associated with hyperglycemia (21, 22)and cardiovascular complications (34, 35) in diabetic patients, as well as with OSA severity (41), underscoring its potential as a prognostic tool in populations with comorbidities. However, while research by Dong et al. validated the predictive value of NLR in patients with diabetes alone, its applicability in diabetes-OSA comorbidity remains insufficiently explored (23). Notably, Chen et al. (42)demonstrated the predictive value of NLR for mortality in prediabetic patients; however, we failed to replicate this association in prediabetic individuals with OSA symptoms, suggesting that comorbid OSA may obscure NLR’s prognostic utility through complex inflammatory crosstalk. The mechanisms involved need to be further studied.

Subgroup analyses revealed significant heterogeneity in NLR’s prognostic value. Males exhibited stronger associations (HR=1.17 vs. 1.10 in females), potentially linked to testosterone-driven inflammatory responses (43). Non-Hispanic black patients had the highest risk (HR=1.47), which may be due to genetic and environmental differences between racial groups that influence the expression of the chronic inflammatory response (44).NLR’s predictive power was amplified in hypertensive patients (HR=1.16), likely reflecting compounded inflammatory burden (45). Moderate smokers (HR=1.31) but not heavy smokers had elevated risks, suggesting a threshold effect of tobacco-induced inflammation (46). The closer association of NLR with cardiovascular mortality in younger patients may be related to age-related physiologic differences, including changes in immune response and inflammatory mechanisms, the exact reasons for which need to be further explored. These findings advocate for NLR-guided risk stratification tailored to demographic and clinical profiles. Furthermore, the complex interactions between NLR and stratification variables indicate that NLR functions as a dynamic biomarker shaped by biological factors (e.g., sex hormones, genetics) and environmental modifiers (e.g., smoking). The lack of association in the prediabetes-OSA subgroup further suggests that comorbid OSA may impair NLR’s predictive capacity through hypoxia-immune crosstalk, necessitating dedicated mechanistic investigations.

In a word, our findings extend current knowledge by demonstrating that NLR’s predictive value is particularly pronounced in diabetic patients with comorbid OSA symptoms. This subgroup faces compounded risks from chronic inflammation and intermittent hypoxia, which may accelerate cardiovascular damage. Importantly, our stratified analyses revealed significant interactions between NLR and variables such as age, smoking, and hypertension, suggesting that NLR could guide personalized monitoring in high-risk subgroups. However, certain limitations must be acknowledged in this study. Firstly, due to its cross-sectional nature, the NHANES study was unable to determine causality. Because baseline NLR measurements may not reflect subsequent changes in inflammatory status as a result of treatment or disease progression, future longitudinal studies are necessary to confirm the relationship between dynamic changes in NLR and mortality. Secondly, residual confounding from unmeasured variables (e.g., genetic factors, detailed medication use) may persist despite adjusting for numerous confounders. Future studies should adopt more refined adjustment strategies or randomized controlled trials to further validate these associations. Thirdly, the findings may not generalize to non-U.S. populations, particularly Asian subgroups, as NHANES primarily reflects the U.S. demographic composition with limited representation of Asian individuals (e.g., <5% in our cohort). Racial differences in genetic susceptibility, lifestyle factors, and disease manifestations may influence NLR’s prognostic utility, necessitating validation in diverse ethnic cohorts. Additionally, self-reported lifestyle variables (e.g., smoking, and alcohol use) might introduce recall bias, though NHANES employs rigorous protocols to minimize such errors. Finally, the biological mechanisms underlying the interaction between NLR, diabetes, and OSA remain speculative; experimental studies are needed to explore inflammatory pathways (e.g., IL-6, TNF-α) and autonomic dysfunction.

## 5 Conclusion

In conclusion, our study demonstrates that elevated NLR levels are significantly associated with increased risks of all-cause mortality and cardiovascular mortality in diabetic patients with OSA symptoms. This association may stem from chronic inflammation, oxidative stress, and autonomic dysfunction. Although the cross-sectional design precludes causal inference, these findings underscore NLR’s potential as an accessible biomarker for identifying high-risk individuals. Future studies should explore mechanistic pathways, such as hypoxia-induced cytokine release, AGE-RAGE signaling, and lymphocyte apoptosis, to elucidate NLR’s role in disease progression and its potential therapeutic applications.

## Data Availability

The raw data supporting the conclusions of this article will be made available by the authors, without undue reservation.
